# Grotenhout Virus, a Novel Nairovirus Found in *Ixodes ricinus* in Belgium

**DOI:** 10.1128/genomeA.00288-17

**Published:** 2017-05-25

**Authors:** Bert Vanmechelen, Lies Laenen, Valentijn Vergote, Piet Maes

**Affiliations:** KU Leuven, Rega Institute, Zoonotic Infectious Diseases Unit (ZIDU) Lab, Leuven, Belgium

## Abstract

We report here the draft genome of a novel nairovirus, Grotenhout virus, isolated from deer ticks (*Ixodes ricinus*) in Belgium. The genome consists of two segments, L and S, and is most similar to the tick-borne South Bay virus, with amino acid identities ranging from 60 to 64%.

## GENOME ANNOUNCEMENT

The genus *Nairovirus* forms one of five genera within the family *Bunyaviridae*, a large group of enveloped, negative-sense, single-stranded RNA viruses with a trisegmented genome organization known to infect a wide variety of hosts, ranging from different types of mammals to a plethora of insects (1). Members of the genus *Nairovirus* are predominantly tick-borne viruses and are capable of infecting a range of vertebrate hosts. Although the clinical importance of nairovirus infection remains to be elucidated for most members of this genus, several *Nairovirus* species are known to be involved in human disease, causing differing pathologies that can in some cases be associated with significant mortality (2). We report here the genome sequences of a novel member of the genus* Nairovirus*, Grotenhout virus, isolated from female deer ticks (*Ixodes ricinus*, also known as castor bean ticks or sheep ticks) originating from Belgium.

Ten female ticks caught in the forest of Grotenhout, Belgium, were pooled and subjected to benzonase/micrococcal nuclease treatment and subsequent tissue homogenization using a Minilys homogenizer (Bertin Technologies). Next, the RNA was extracted using the QIAamp Viral RNA Mini Kit (Qiagen). The extracted RNA was amplified by whole-transcriptome amplification (WTA2; Sigma-Aldrich) and subsequently subjected to Illumina NextSeq 500 sequencing (Illumina). A *de novo* assembly was generated using the CLC Genomics Workbench (version 9.5.2; Qiagen) by coassembling both paired and unpaired reads. A tBLASTx search of the generated contigs identified 13 contigs, composed of 3,783 reads, that displayed significant similarity to South Bay virus, an unclassified nairovirus (3). Merging of these contigs was done using SeqMan (version 7.0.0; DNAStar). PCR amplification, using the OneStep RT-PCR kit (Qiagen), followed by Sanger sequencing on an ABI Prism 3130xl genetic analyzer (Applied Biosystems), was used to close the remaining gaps.

The genome sequence of Grotenhout virus consists of two segments 14,848 and 3,578 nucleotides in length, representing the L and S segments, respectively. Phylogenetic clustering of this virus with other members of the family *Bunyaviridae*, based on the coding sequence of the L segment, groups this virus within the genus *Nairovirus*, with South Bay virus being the most closely related virus. The L segment contains a 4,812-amino acid (aa) polymerase gene displaying 64% amino acid similarity with South Bay virus, while the S segment contains a 551-aa nucleocapsid gene (60% similarity with South Bay virus). Members of the family *Bunyaviridae*, including those belonging to the genus *Nairovirus*, typically have a third genomic segment, the M segment, which encodes two or more structural glycoproteins. Despite the use of extensive bioinformatics-based search strategies, a putative M segment for Grotenhout virus could not be identified, something also observed for South Bay virus (3). Whether the absence of an identifiable M segment is due to these viruses actually having only two segments or due to the M segment of these viruses being so different from known nairovirus M segments that it is practically impossible to identify them as such remains to be established.

### Accession number(s).

The genome sequence of Grotenhout virus has been deposited in NCBI GenBank under the accession numbers KY700683 (S segment, complete coding region) and KY700684 (L segment, complete segment sequence).
